# P-238. Revolutionizing Infection Prevention: Using Kamishibai Chart in Digital Format for Rehabilitation Hospital Advancement

**DOI:** 10.1093/ofid/ofae631.442

**Published:** 2025-01-29

**Authors:** Tatiane Barbosa Mendes De Freitas Lemes, Ludmila Gomes dos Santos Oliveira, Ronycley Resende Rocha, Fabianne Silveira Cardoso, Adriana Oliveira Guilarde, Lucas Candido Gonçalves Barbosa, Ariana Rocha Romão Godoi, Ciro Bruno Silveira Costa, Lísia Gomes Martins de Moura Tomich

**Affiliations:** Centro Estadual de Reabilitação e Readaptação Dr. Henrique Santillo, Goiânia, Goias, Brazil; Centro Estadual de Reabilitação e Readaptação Dr. Henrique Santillo, Goiânia, Goias, Brazil; Centro Estadual de Reabilitação e Readaptação Dr. Henrique Santillo, Goiânia, Goias, Brazil; Centro Estadual de Reabilitação e Readaptação Dr. Henrique Santillo, Goiânia, Goias, Brazil; physiotherapist, Goiania, Goias, Brazil; Centro Estadual de Reabilitação e Readaptação Dr. Henrique Santillo, Goiânia, Goias, Brazil; Centro Estadual de Reabilitação e Readaptação Dr. Henrique Santillo, Goiânia, Goias, Brazil; Centro Estadual de Reabilitação e Readaptação Dr. Henrique Santillo, Goiânia, Goias, Brazil; Centro Estadual de Reabilitação e Readaptação Dr. Henrique Santillo, Goiânia, Goias, Brazil

## Abstract

**Background:**

HAIs are a significant public health concern in Brazil, leading to higher hospital costs and increased rates of morbidity and mortality. This study evaluated the effectiveness of implementing the mechanical ventilation (MV) maintenance bundle in preventing ventilator-associated pneumonia (VAP) within an ICU, by comparing outcomes before and after the introduction of the Kamishibai chart strategy in electronic format.

Kamishibai Board - mechanical ventilation maintenance bundles on the dynamic television panel in the ICU

**Figure:**
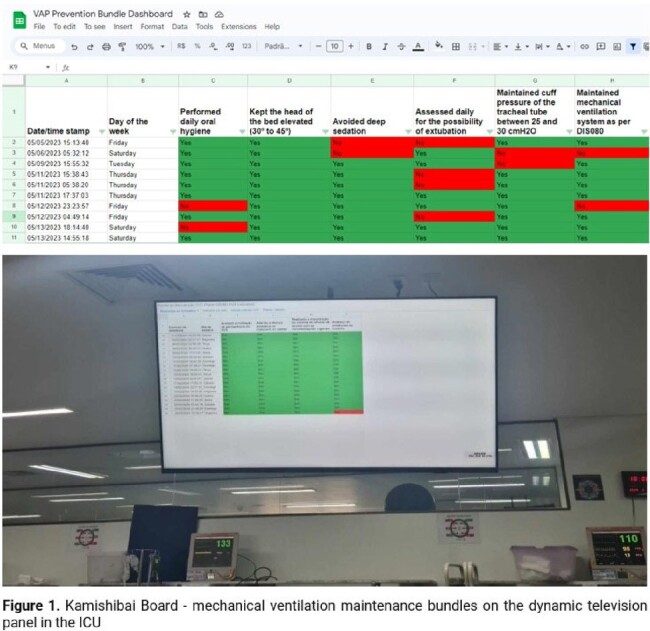

**Methods:**

The Crer in Brazil, is a top-tier rehabilitation-focused hospital with a 20-bed ICU. From August 2020 to April 2022, adherence to the MV maintenance bundle was assessed using an "all or nothing" compliance approach. This was compared from May 2022 to July 2023, when a detailed assessment method was introduced. Each component was checked daily by an ICU team using Google Forms, feeding data into an electronic dashboard resembling a Kamishibai board for real-time management and resolution of non-conformities. The VAP Incidence Density (ID) indicator was calculated and statistical analysis included the Shapiro-Wilk test, Student's t-test and Winters test to analyze trends and forecasts before bundle implementation (p < 0.05).

Comparison of mean adherence to the mechanical ventilator maintenance bundle for prevention of VAP

**Figure:**
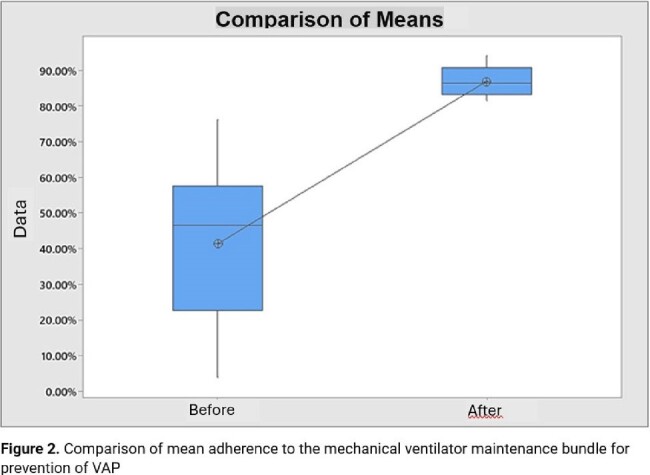

**Results:**

After modifying the criteria for assessing adherence by items, there was an improvement in the adherence rate to the MV maintenance bundle. The mean adherence rate during the initial period was 41.5%, which significantly increased to 86.9% afterward (mean difference of 45%; p< 0.001). Prior to the intervention, the average VAP ID was 7.0±4.50, while post-intervention, it decreased to 1.5±3.12, reflecting an average reduction of 5.53; p< 0.001. Furthermore, the average monthly rate of VAP decreased from 2.38 ±2.38 before the intervention to 0.26±0.59 afterward, demonstrating an average reduction of 2.11; p=0.001. The average duration of MV days decreased from 309±167 before the intervention to 172±30.4 afterward, resulting in an average reduction of 137 days; p=0.001. The Winters test confirmed the efficacy of the intervention by simulating a scenario without action.

Winters Test: Predictions of ventilator-associated pneumonia incidence density if no action were taken (95% CI).

**Figure:**
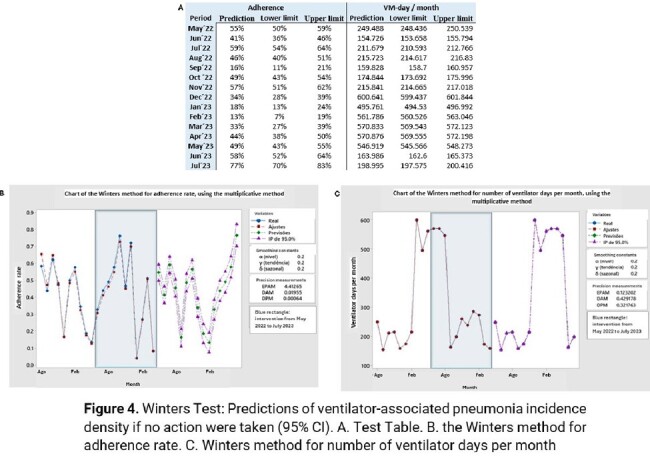

A. Test Table. B. the Winters method for adherence rate. C. Winters method for number of ventilator days per month

**Conclusion:**

Implementing the structured bundle in the ICU with the electronic Kamishibai chart significantly improved adherence to the MV maintenance bundle, suggesting potential indirect cost savings by preventing HAIs and reducing HAI-related mortality.

**Disclosures:**

**All Authors**: No reported disclosures

